# More Favorable Short and Long-Term Outcomes for Screen-Detected Colorectal Cancer Patients

**DOI:** 10.3389/fonc.2021.620644

**Published:** 2021-03-15

**Authors:** Gaya Spolverato, Giulia Capelli, Jessica Battagello, Andrea Barina, Susi Nordio, Elena Finotti, Isabella Mondi, Corrado Da Lio, Emilio Morpurgo, Josè Adolfo Navarro, Fabio Ceccato, Alessandro Perin, Corrado Pedrazzani, Giulia Turri, Giacomo Zanus, Michela Campi, Marco Massani, Adriana Di Giacomo, Daniela Prando, Ferdinando Agresta, Salvatore Pucciarelli, Manuel Zorzi, Massimo Rugge

**Affiliations:** ^1^ Department Surgical, Oncological and Gastroenterological Sciences, University of Padova, Padova, Italy; ^2^ Veneto Tumor Registry, Azienda Zero, Padova, Italy; ^3^ General Surgery Unit, “SS. Giovanni e Paolo” Hospital, Venezia, Italy; ^4^ General Surgery Unit, Mirano Hospital, Mirano, Italy; ^5^ General Surgery Unit, Camposampiero Hospital, Camposampiero, Italy; ^6^ General Surgery Unit, “Sant’Antonio” Hospital, Padova, Italy; ^7^ Division of General and Hepatobiliary Surgery, Department of Surgical Sciences, Dentistry, Gynecology and Pediatrics, University of Verona, Verona, Italy; ^8^ IV Surgical Unit, Ca’ Foncello Hospital, Treviso, Italy; ^9^ III Surgical Unit, Ca’ Foncello Hospital, Treviso, Italy; ^10^ Department of General Surgery, Adria Civil Hospital, Adria, Italy; ^11^ Department of Medicine DIMED, Pathology and Cytopathology Unit, University of Padova, Padova, Italy

**Keywords:** colorectal cancer, surgery, screening, colorectal cancer outcomes, disease free survival, overall survival

## Abstract

**Background:**

Screening significantly reduces mortality from colorectal cancer (CRC). Screen detected (SD) tumors associate with better prognosis, even at later stage, compared to non-screen detected (NSD) tumors. We aimed to evaluate the association between diagnostic modality (SD vs. NSD) and short- and long-term outcomes of patients undergoing surgery for CRC.

**Materials and Methods:**

This retrospective cohort study involved patients aged 50–69 years, residing in Veneto, Italy, who underwent curative-intent surgery for CRC between 2006 and 2018. The clinical multi-institutional dataset was linked with the screening dataset in order to define diagnostic modality (SD vs. NSD). Short- and long-term outcomes were compared between the two groups.

**Results:**

Of 1,360 patients included, 464 were SD (34.1%) and 896 NSD (65.9%). Patients with a SD CRC were more likely to have less comorbidities (p = 0.013), lower ASA score (p = 0.001), tumors located in the proximal colon (p = 0.0018) and earlier stage at diagnosis (p < 0.0001). NSD patients were found to have more aggressive disease at diagnosis, higher complication rate and higher readmission rate due to surgical complications (all p < 0.05). NSD patients had a significantly lower Disease Free Survival and Overall Survival (all p < 0.0001), even after adjusting by demographic, clinic-pathological, tumor, and treatment characteristics.

**Conclusions:**

SD tumors were associated with better long-term outcomes, even after multiple adjustments. Our results confirm the advantages for the target population to participate in the screening programs and comply with their therapeutic pathways.

## Introduction

Colorectal cancer (CRC) represents one of the leading causes of cancer death in Western countries. In the United States, it represents the third most common cancer in both men and women, with about 147,000 cases estimated in 2020 (1). A similar figure can be identified in Europe, where CRC accounted for more than 242,000 estimated deaths in 2018 (2). Variability in the incidence and mortality rates between different countries can be attributed to lifestyle risk factors, as well as to the presence and availability of screening programs (3).

Screening based on the Fecal Occult Blood Test (FOBT) has been shown to significantly reduce the risk of mortality from CRC (3–6). Compared to endoscopy, FOBT obtained a higher acceptance from the general population (ranging from 50% to 70% in the European trials) (7–9). Acknowledging this, in 2003 the European Council recognized FOBT as a valuable and effective screening method and recommended the implementation of organized CRC screening in all European countries (10). During the following years, the higher sensitivity and specificity of fecal immunochemical testing (FIT) compared with guaiac FOBT for the detection of advanced adenomas and CRC was established (11), yielding a high effectiveness in reducing mortality as well as incidence of CRC (12, 13).

Screen detected (SD) tumors tend to be at earlier stages compared to non-screen detected (NSD) ones, with a favorable impact on patients’ prognosis (4). Moreover, it has been noticed that screened patients’ survival advantage remains consistent even with later stage tumors (14–16). Several explanations have been offered for this phenomenon: for instance, it has been supposed that SD cancers had more favorable genetic characteristics, leading to a slower growth and a higher chance to be identified by screening. Besides, patients who are adherent to screening programs may be more health-conscious and more compliant with therapy (14). Nevertheless, to our knowledge, no study has offered univocal and evidence-based explanation of the mechanisms underlying such evidence.

In 2018, the Dutch ColoRectal Audit investigated the impact of FIT–based screening program on surgical outcomes of patients treated for CRC in the Netherlands (17). This study confirmed that patients whose cancer was SD had better outcomes, even after an extensive case-mix correction. However, these results need to be confirmed in other countries where a screening program is ongoing.

The objective of the current study was to evaluate the impact of diagnostic modality (SD vs. NSD) on short-term outcomes (i.e., complications, re-interventions), long-term outcomes (i.e., disease-free survival, overall survival), and on the quality of the therapeutic pathway of patients undergoing surgery for CRC.

## Materials And Methods

### Setting

The current study was carried out in the Veneto region, Italy, where a CRC screening program based on FIT has been operating since 2002. The target population includes residents aged 50–69 years, who are invited to complete a FIT every two years. Subjects with a positive FIT (>20 µg Hb/g feces) are contacted to undergo a colonoscopy, which is performed at an endoscopic referral center. Patients are successively referred for surgery, post-colonoscopy surveillance, or further rounds of FIT, depending on the colonoscopy results. The FIT test, colonoscopy, and surgery are free of charge. For the purposes of this study, all patients with a CRC diagnosed at colonoscopy following a positive screening FIT were considered Screen detected (SD), while all other CRC patients in the same age range were considered as non-screen detected (NSD).

### Patients Selection

All patients aged 50–69 years, residing in Veneto, who underwent curative-intent surgery for histologically confirmed CRC between 2006 and 2018 in nine participating Surgery Units were identified. Low-, middle-, and high-volume centers were included in order to obtain data that are more generalizable. Centers provided data for the entire study period or for a part of it, ranging from 3 to 13 years. No selections were operated. The range of patients enrolled for each center was 17–372.

Urgent and emergency cases, defined according to the definition of Acute Care Surgery (ACS) provided by the American Association for the Surgery of Trauma (AAST) Committee on Severity Assessment and Patient Outcomes (18), were excluded to minimize confounding bias.

Data on demographic, clinic-pathological, tumor, and treatment characteristics were collected. Resection margin status was classified as microscopically negative (R0), microscopically positive (R1), or macroscopically positive (R2). Perioperative data, such as length of stay (LOS), estimated blood loss (EBL), transfusions, complications, readmission, reoperation, and mortality were collected. Perioperative complications, distinguished between surgical and medical, were considered within 30 days from the operation and were defined according to the Clavien-Dindo classification (19). Re-admissions and reoperations were considered within 30 days after surgery, while mortality was recorded as in-hospital or within 30 days. Readmissions were classified as “surgery-related” and “non- surgery-related”. Finally, data on local and distant recurrence were reported. Patients’ follow-up was conducted according to the Guidelines on Colorectal Cancer of the Italian Association of Medical Oncology (AIOM) (20). In detail, a colonoscopy was performed 1 year after surgery; if negative, endoscopic surveillance was scheduled 3 years after surgery, and then every 5 years. Patients underwent a chest and abdomen CT scan every 6–12 months for the first 3–5 years too. Finally, a clinical examination with digital rectal examination and measurement of Carcino-Embryonic Antigen (CEA) levels were performed every 6 months for the first 5 years after surgery.

The clinical multi-institutional dataset was linked with the screening program dataset in order to define the diagnostic modality (SD vs. NSD). A further linkage with the regional Hospital Discharge Database and the regional Outpatient Service Database was used to determine 30-day readmissions, reoperations, adjuvant treatments, and to complete missing data on tumor recurrence.

The vital status of all subjects was assessed through record linkage with the population file of residents, as available from the regional Healthcare System, and with the regional Mortality Registry (available up to December 31^st^ 2018).

### Statistical Analysis

Descriptive statistics were used to summarize the main characteristics of the two study groups (SD and NSD) and differences in categorical variables distribution were tested using the Chi-square test.

Multivariate logistic regression analyses were performed to estimate the adjusted odds ratios for quality of the therapeutic pathway and short-term outcomes. Differences between SD and NSD were adjusted using the following explanatory variables: gender (female, male), age (50–59, 60–69 years), tumor stage, cancer site (colon, rectum, not specified or multiple), surgical approach (mini-invasive: laparoscopic, robotic, and local excision; invasive: laparotomy), stoma creation (yes, no), comorbidity (yes, no), surgery unit (categorized into hubs and spokes), neoadjuvant treatment (yes, no), adjuvant treatment (yes, no), resection margin status (R0, R1, R2), lymphatic invasion (yes, no), vascular invasion (yes, no), perineurial invasion (yes, no), and grading (G1, G2, G3, G4).

The survival outcomes included overall (OS) and disease-free survival (DFS), defined as the time interval between the date of surgery and the date of death or recurrence, respectively. Time was censored at the date of last follow-up. In order to compare the OS and DFS of the two study groups, adjusted hazard ratios were calculated using Cox-Regression Models. The survival distributions of the two groups were compared through the log-rank test.

A p-value less than 0.05 was considered statistically significant. Statistical analyses were performed using SAS, version 9.4 (SAS Institute, Cary, NC) and R-software environment (R Core Team, Vienna).

### Ethics

The Italian legislation identifies Cancer Registries as collectors of personal data for surveillance purposes without explicit individual consent. The approval of a research ethics committee was not required, since this study is a descriptive analysis of individual data without any direct or indirect intervention on patients (21).

## Results

### Patients Clinic-Pathological Characteristics

A total of 1,449 patients aged 50–69 years underwent surgery for CRC in the index hospitals. Eighty-nine (6.1%) were excluded because they were not resident in the Veneto region. Among the remaining 1,360 patients, 464 were SD (34.1%) and 896 NSD (65.9%), respectively. The characteristics of study population are summarized in **Table 1**.

**Table 1:** Main characteristics of the study population.

Variable		Overall		Non screen detected		Screen detected		p-value°
		N	%*	N	%*	N	%*	
Total (row %)		1,360	100	896	65.9	464	34.1	
Year of diagnosis	2006–2009	71	5.2	67	7.5	4	0.9	<0.0001
	2010–2013	595	43.8	377	42.1	218	47	
	2014–2018	694	51	452	50.4	242	52.2	
Sex	Men	803	59	539	60.2	264	56.9	0.25
	Women	557	41	357	39.8	200	43.1	
Age (years)	50–59	518	38.1	332	37.1	186	40.1	0.27
	60–69	842	61.9	564	62.9	278	59.9	
ASA score	1–2	691	82.9	429	79.3	262	89.4	0.0001
	3–4	143	17.1	112	20.7	31	10.6	
	*Missing*	*526*	*(38.7)*	*355*	*(39.6)*	*171*	*(36.9)*	
Body Mass Index	<25	438	43.9	317	45.8	121	39.7	0.19
	25–30	398	39.9	268	38.7	130	42.6	
	≥30	161	16.1	107	15.5	54	17.7	
	*Missing*	*363*	*(26.7)*	*204*	*(22.8)*	*139*	*(30.0)*	
Comorbidities	Yes	583	59.7	411	62.4	172	54.1	0.0134
	No	394	40.3	248	37.6	146	45.9	
	*Missing*	*383*	*(28.2)*	*237*	*(26.5)*	*146*	*(31.5)*	
Tumor Site	Colon, proximal	491	36.2	298	33.3	193	41.9	0.0018
	Colon, distal	422	31.1	276	30.9	146	31.7	
	Rectum	418	30.8	298	33.3	120	26	
	Not specified or multiple	24	1.8	22	2.5	2	0.4	
	*Missing*	*5*	*(0.4)*	*2*	*(0.2)*	*3*	*(0.6)*	
pTNM staging	0	83	6.5	52	6.3	31	6.9	<0.0001
	1	400	31.3	189	22.8	211	47.1	
	2	270	21.2	179	21.6	91	20.3	
	3	324	25.4	228	27.5	96	21.4	
	4	199	15.6	180	21.7	19	4.2	
	*Missing *	*84*	*(6.2)*	*68*	*(7.6)*	*16*	*(3.4)*	
Grading	1	114	12.4	62	10.2	52	16.9	0.0004
	2	641	70	418	68.8	223	72.4	
	3	160	17.5	127	20.9	33	10.7	
	4	1	0.1	1	0.2	0	0	
	*Missing*	*444*	*(32.6)*	*288*	*(32.1)*	*156*	*(33.6)*	
Lymphatic invasion	Yes	223	32.1	163	36.2	60	24.6	0.0017
	No	471	67.9	287	63.8	184	75.4	
	*Missing*	*666*	*(49.0)*	*446*	*(49.8)*	*220*	*(47.4)*	
Vascular invasion	Yes	417	48.5	306	54.2	111	37.8	<0.0001
	No	442	51.5	259	45.8	183	62.2	
	*Missing*	*501*	*(36.8)*	*331*	*(36.9)*	*170*	*(36.6)*	
Perineurial invasion	Yes	259	29.1	205	35.2	54	17.6	<0.0001
	No	631	70.9	378	64.8	253	82.4	
	*Missing*	*470*	*(34.6)*	*313*	*(34.9)*	*157*	*(33.8)*	
Resection margin status	R0	1186	89.8	754	86.8	432	95.6	<0.0001
	R1	23	1.7	20	2.3	3	0.7	
	R2	112	8.5	95	10.9	17	3.8	
	*Missing*	*39*	*(2.9)*	*27*	*(3.0)*	*12*	*(2.6)*	
Neoadjuvant therapy	Yes (rectum)	182	14.3	152	18.4	30	6.7	<0.0001
	Yes (colon)	44	3.5	42	5.1	2	0.4	
	Yes (not specified or multiple)	3	0.2	3	0.4	0	0	
	No	1042	82	628	76.1	414	92.8	
	*Missing*	*89*	*(6.5)*	*71*	*(7.9)*	*18*	*(3.9)*	
Adjuvant therapy	Yes	524	47.5	381	55.5	143	34.3	<0.0001
	No	579	52.5	305	44.5	274	65.7	
	*Missing*	*257*	*(18.9)*	*210*	*(23.4)*	*47*	*(10.1)*	

*Percentages are computed on patients with available data. Percentage of missing data are computed on all patients.

°χ2 test was used.

ASA, American Society of Anesthesiologists.

Overall, 803 cases were men (59%) and 557 women (41%); most patients (n = 842, 61.9%) were 60–69 years old, with an ASA score of 1-2 (n = 691, 82.9), and at least one comorbidity (n = 583, 59.7%). Body mass index (BMI) was less than 30 in 83.3% (n = 836) of patients. The majority of tumors were located in the proximal colon (n = 491, 36.2%) and were TNM stage I at diagnosis (n = 400, 31.3%). Most patients underwent surgery between 2014 and 2018 (n = 694, 51.0%).

Compared with patients in the NSD group, those in the SD group were more likely to have less comorbidities (p = 0.013), lower ASA score (p = 0.001), tumors located in the proximal colon (p = 0.0018), and earlier stage at diagnosis (p < 0.0001).

### Peri-Operative and Surgical Treatment Characteristics

Overall, the median operative time was 234.7 min. The most frequently performed operation was right hemicolectomy (n = 446, 32.9%), followed by rectal anterior resection (n = 272, 20.1%) and sigmoidectomy (n = 223, 16.5%). Only 24 (1.8%) patients underwent transanal surgery. The remaining patients underwent either left hemicolectomy (n = 132, 9.7%), segmental colonic resection (n = 128, 9.5%), non- specified rectal resection (n = 83, 6.1%) or abdominoperineal resection (n = 46, 3.4%). Details about surgical intervention were not reported for six patients.

The majority of patients were either treated with a invasive approach (n = 808, 65.2%) and/or avoided the stoma creation (n = 1056, 77.9%). Intraoperative blood loss >500 ml occurred in 222 (22.6%) patients, and 108 (10.2%) required perioperative blood transfusions. As for histopathologic findings, most patients had a single neoplasia (n = 742, 97.5%), usually an adenocarcinoma (n = 1,244, 92.9%). Most patients had a low histopathologic grade (1, 2) (n = 755; 82.4%) and a low stage at histopathologic examination (i.e., stage I- II) (n = 753, 59.0%). The mean number of sampled lymph nodes was 18.31 (SD 11.41), with a mean rate of positivity of 9.21% (SD 0.18); a total number of 1,186 (89.8%) patients underwent a R0 resection.

Again, differences were found between the two groups. Rectal resection was more often performed in the NSD group (NSD: n = 248, 27.8% vs. SD: n = 107, 23.2%; p = 0.0034), reflecting a higher percentage of rectal tumors in this group of patients. Patients in the NSD group were also more likely to undergo open surgery (NSD: n = 334, 42.0% vs. SD: n = 98, 22.0%; p < 0.0001) and to require a stoma (NSD: n = 234, 26.2% vs. SD: n = 65, 14.1%; p < 0.0001). EBL >500ml (NSD: n = 185, 30.4% vs. SD: n = 37, 9.9%; p < 0.0001) and transfusions (NSD: n = 91, 13.5% vs. SD: n = 17, 4.3%; p < 0.0001) were also more common in NSD patients. As for histopathology, NSD patients had a higher risk to undergo an R1-2 resection (NSD: n = 115, 13.2% vs. SD: n = 20, 4.4%; p < 0.0001). Patients in the NSD group also had a higher rate of perineurial (NSD: n = 205, 35.2% vs. SD: n = 54, 17.6%; p < 0.0001), vascular (NSD: n = 306, 54.2% vs. SD: n = 111, 37.8%; p < 0.0001) and lymphatic invasion (NSD: n = 163, 36.2% vs. SD: n = 60, 24.6%; p = 0.00017). Moreover, pathological stage III-IV were more likely in NSD patients (NSD: n = 408, 49.3% vs. SD: n = 115, 25.7%; p < 0.0001).

### Short-Term Postoperative Outcomes

Morbidity and short-term outcomes after surgery are summarized in **Table 2**. Postoperative complications occurred in 369 patients (27.1%). Most patients experienced minor complications (i.e., Clavien-Dindo 1-2). Surgical complications were more common than medical complications (n = 250, 18.4% vs. n = 121, 8.9%, respectively) and included bleeding (n = 111, 8.2%), anastomotic leakage (n = 77, 5.7%), prolonged ileum or small bowel obstruction (n = 25, 1.8%) and surgical site infection (n = 63, 4.6%). Medical complications included renal or urinary (n = 13, 1%), respiratory (n = 17, 1.3%), cardiovascular (n = 7, 0.5%), and cerebrovascular (n = 7, 0.5%) complications. Median LOS was 9 days. Mortality at 30 days was 0.2% (n = 3). Patients in the NSD group had a higher complication rate overall (NSD: n = 263, 29.4% vs. SD: n = 106, 22.8%; p = 0.01). The excess was confirmed comparing the rate of major complications (NSD: n = 43, 10% vs. SD: n = 18, 7.9%; p = 0.02). Surgical complications were (non-significantly) less common in SD patients (OR 0.76, 95% CI 0.44–1.34). Among surgical complications, bleeding occurred significantly more often in the NSD group (NSD: n = 93, 10.4% vs. SD: n = 18, 3.9%; p < 0.0001). As for medical complications, no significant difference could be found between the two groups. Median LOS was significantly higher in the NSD group (NSD: 10 days vs. SD: 9 days; p < 0.0001). As for mortality, two of the three patients who died within 30 days from intervention were in the NSD group.

**Table 2:** Morbidity and short-term outcomes after surgery.

Variable		Overall		Non screen detected		Screen detected		p-value°
		N	%	N	%	N	%	
Patients with any complication		369	27.1	263	29.4	106	22.8	0.01
Complications according to Clavien-Dindo Classification	0	290	44.0	169	39.1	121	53.3	0.0005
	1–2	308	46.7	220	50.9	88	38.8	0.0029
	3–5	61	9.3	43	10.0	18	7.9	0.02
Complication type	Patients with at least 1 surgical complication	250	18.4	185	20.6	65	14.0	0.0027
	Patients with at least 1 medical complication	121	8.9	82	9.2	39	8.4	0.65
Surgical complications	Anastomotic leak/dehiscence	77	5.7	49	5.5	28	6.0	0.67
	Prolonged ileus/Small Bowel Obstruction	25	1.8	19	2.1	6	1.3	0.28
	Surgical site infection	63	4.6	43	4.8	20	4.3	0.68
	Bleeding	111	8.2	93	10.4	18	3.9	<0.0001
	Other	4	0.3	3	0.3	1	0.2	0.70
Medical complications	Renal/urinary	13	1.0	9	1.0	4	0.9	0.80
	Respiratory	17	1.3	11	1.2	6	1.3	0.92
	Cardiovascular	7	0.5	6	0.7	1	0.2	0.27
	Cerebrovascular	7	0.5	7	0.8	0	0.0	0.056
	Other	91	6.7	63	7.0	28	6.0	0.49
Lenght of hospital stay in days (median)		9		10		9		<0.0001*
In-hospital mortality		3	0.2	3	0.3	0	0.0	<0.0001
30 days mortality		3	0.2	2	0.2	1	0.2	0.97

°χ2 test was used.

*Mann-Whitney test.

Readmission and reoperation rates at 30 days are reported in **Table 3**.

**Table 3:** Readmission and reoperation rates at 30 days.

Variable		Overall		Non screen detected		Screen detected		p-value°
		N	%	N	%	N	%	
30 days reoperation	Patients with at least 1 reoperation	194	14.3	144	16.1	50	10.8	0.008
	Patients with at least 1 surgery- related reoperation	95	7.0	70	7.8	25	5.4	0.10
	Patients with at least 1 surgical complication	71	5.2	51	5.7	20	4.3	0.28
	Anastomotic leak/dehiscence	35	2.6	21	2.3	14	3.0	0.46
	Prolonged ileus/Small Bowel Obstruction	18	1.3	15	1.7	3	0.6	0.12
	Surgical site infection	18	1.3	14	1.6	4	0.9	0.28
	Bleeding	3	0.2	2	0.2	1	0.2	0.98
	Other	2	0.1	1	0.1	1	0.2	0.64
	Patients with at least 1 medical complication	36	2.6	28	3.1	8	1.7	0.13
	Renal/urinary	6	0.4	5	0.6	1	0.2	0.37
	Respiratory	3	0.2	3	0.3	0	0.0	0.21
	Cardiovascular	3	0.2	3	0.3	0	0.0	0.21
	Cerebrovascular	5	0.4	5	0.6	0	0.0	0.11
	Other	28	2.1	21	2.3	7	1.5	0.30
30 days readmission	Patients with at least 1 readmission	135	9.9	104	11.6	31	6.7	0.004
	Patients with at least 1 surgery- related readmission	109	8.0	84	9.4	25	5.4	0.010
	Patients with at least 1 surgical complication	49	3.6	36	4.0	13	2.8	0.25
	Anastomotic leak/dehiscence	8	0.6	5	0.6	3	0.6	0.84
	Prolonged ileus/Small Bowel Obstruction	16	1.2	11	1.2	5	1.1	0.81
	Surgical site infection	20	1.5	14	1.6	6	1.3	0.70
	Bleeding	5	0.4	4	0.4	1	0.2	0.50
	Other	4	0.3	3	0.3	1	0.2	0.70
	Patients with at least 1 medical complication	43	3.2	32	3.6	11	2.4	0.23
	Renal/urinary	9	0.7	6	0.7	3	0.6	0.96
	Respiratory	6	0.4	5	0.6	1	0.2	0.37
	Cardiovascular	6	0.4	5	0.6	1	0.2	0.37
	Cerebrovascular	6	0.4	6	0.7	0	0.0	0.077
	Other	27	2.0	21	2.3	6	1.3	0.19

°χ2 test was used.

A surgical reintervention was required in 194 patients (14.3%). Among these patients, 71 (5.2%) had at least one surgical complication. The most frequent complication requiring reintervention was anastomotic leakage (n = 35, 2.6%), followed by bowel occlusion (n = 18, 1.3%), surgical site infection (n = 18, 1.3%) and hemorrhage (n = 3, 0.2%). Overall, 135 patients (9.9%) were readmitted within 30 days from intervention, while 109 (8.0%) experienced a surgery- related readmission. Of these patients, 49 (3.6%) had at least one surgical complication, mainly surgical site infection (n = 20, 1.5%), bowel occlusion (n = 16, 1.2%) and anastomotic leakage (n = 8, 0.6%). Among the 194 patients who underwent a reoperation, 144 were in the NSD group (74.2%); when considering surgery-related reoperations, these patients had a higher but not significant risk compared to SD patients. As for 30-day readmission, out of the 135 readmitted patients, 104 were from the NSD group (77.0%); this difference could be observed even considering only surgery- related readmissions (NSD: n = 84, 9.4% vs. SD: n = 25, 5.4%; p = 0.01).

### Work Up Quality Indicators

Work up quality indicators are summarized in **Table 4**. According to the adjusted logistic regression, the diagnosis-to-treatment (surgery or neoadjuvant therapy, whichever came first) time was shorter in the NSD group. Patients in the SD group had an adjusted OR of 0.55 (95% CI 0.40– 0.77) to be treated within 30 days from diagnosis. Conversely, patients in the SD group had a shorter waiting time from surgery to adjuvant chemotherapy (OR of being treated within 8 weeks 2.20, 95% CI 1.21–4.03). A clinically relevant EBL was significantly lower in the SD group (OR 0.31; 95% CI 0.12–0.80).

**Table 4:** Adjusted odds ratios of the quality of the therapeutic pathway and of short-term outcomes, and adjusted hazard ratios of disease free- and overall survival by diagnostic modality, with 95% confidence intervals (CI).

Category	Outcome	Adjusted^1^ Odds Ratio (SD vs. NSD) (95% CI)	p-value
1. Quality of the therapeutic pathway	1.1: Time between diagnosis and treatment ≤ 30 days	0.55 (0.40–0.77)^1^	0.0004
	1.2: Time between surgery and chemotherapy < 8 weeks	2.20 (1.21–4.03)^2^	0.01
	1.3: Length of postoperative hospital stay ≤ 12 days	1.16 (0.70–1.93)^2^	0.56
2. Short-term outcomes	2.1: 30 days readmission	0.65 (0.32–1.32)^3^	0.23
	2.2: 30 days surgery- related readmission	0.77 (0.35–1.66)^3^	0.51
	2.3: 30 days reintervention	0.72 (0.40–1.29)^3^	0.27
	2.4: 30 days surgery- related reintervention	0.84 (0.35–2.00)^3^	0.69
	2.5: EBL >500 ml	0.31 (0.12–0.80)^3^	0.01
	2.6: Readmission due to surgical complications	0.96 (0.32–3.02)^3^	0.98
	2.7: Readmission due to medical complications	1.15 (0.36–3.65)^3^	0.81
	2.8: Reinterventions due to surgical complications	1.87 (0.72–4.84)^3^	0.46
	2.9: Reinterventions due to medical complications	0.84 (0.24–2.95)^3^	0.78
	2.10: Surgical complications	0.76 (0.44–1.34)^3^	0.34
	2.11: Medical complications	1.18 (0.57–2.44)^3^	0.66
	2.12: Complications (surgical or medical)	0.87 (0.53–1.41)^3^	0.56
3. Survival	3.1: Disease Free Survival	0.40 (0.22–0.73)^4^	0.0029
	3.2: Overall Survival	0.25 (0.12–0.51)^4^	0.0002

^1^According to logistic regression adjusted by gender, age, CRC stage at diagnosis, cancer site, surgery unit, and comorbidity.

^2^According to logistic regression adjusted by gender, age, CRC stage at diagnosis, cancer site, surgery unit, comorbidity, surgical approach, stoma creation, and neoadjuvant treatment.

^3^According to logistic regression adjusted by gender, age, CRC stage at diagnosis, cancer site, surgery unit, comorbidity, surgical approach, stoma creation, neoadjuvant treatment, resection margin, lymphatic invasion, vascular invasion, perineurial invasion, and grading.

^4^According to Cox regression model adjusted by gender, age, CRC stage at diagnosis, cancer site, surgery unit, comorbidity, surgical approach, stoma creation, neoadjuvant treatment, resection margin, lymphatic invasion, vascular invasion, perineurial invasion, grading, and adjuvant treatment.

SD, screen detected; NSD, non screen detected; EBL, Estimated Blood Loss.

When analyzing only patients with a cancer located in the colon, a screening diagnosis was associated with a significant reduction of 30-day readmission rate (OR 0.33; 95% CI 0.12–0.93), of surgical complications (OR 0.49; 95% CI 0.23–1.00) and of overall complications (OR 0.54; 95% CI 0.29–0.99) (**Supplementary Table 1**). On the other hand, no statistically significant differences in the quality of the therapeutic pathway and short-term indicators were observed among patients with rectal cancer, likely due to the low number of cases (**Supplementary Table 2**).

### Long-Term Outcomes

**Figure 1:**
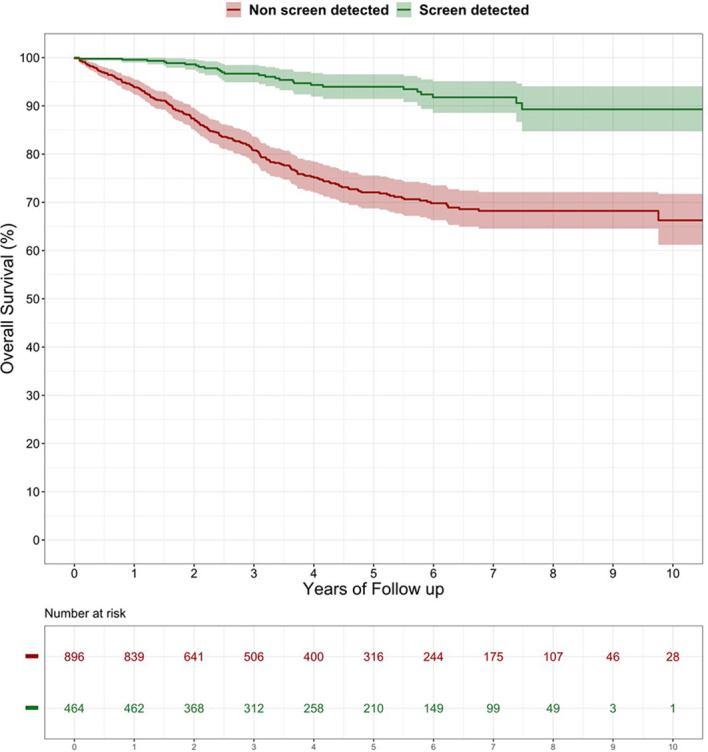
Disease-free survival after surgery among patients with screen detected versus non-screen detected colorectal cancer (%). Log-rank test: p-value < 0.0001.

**Figure 2:**
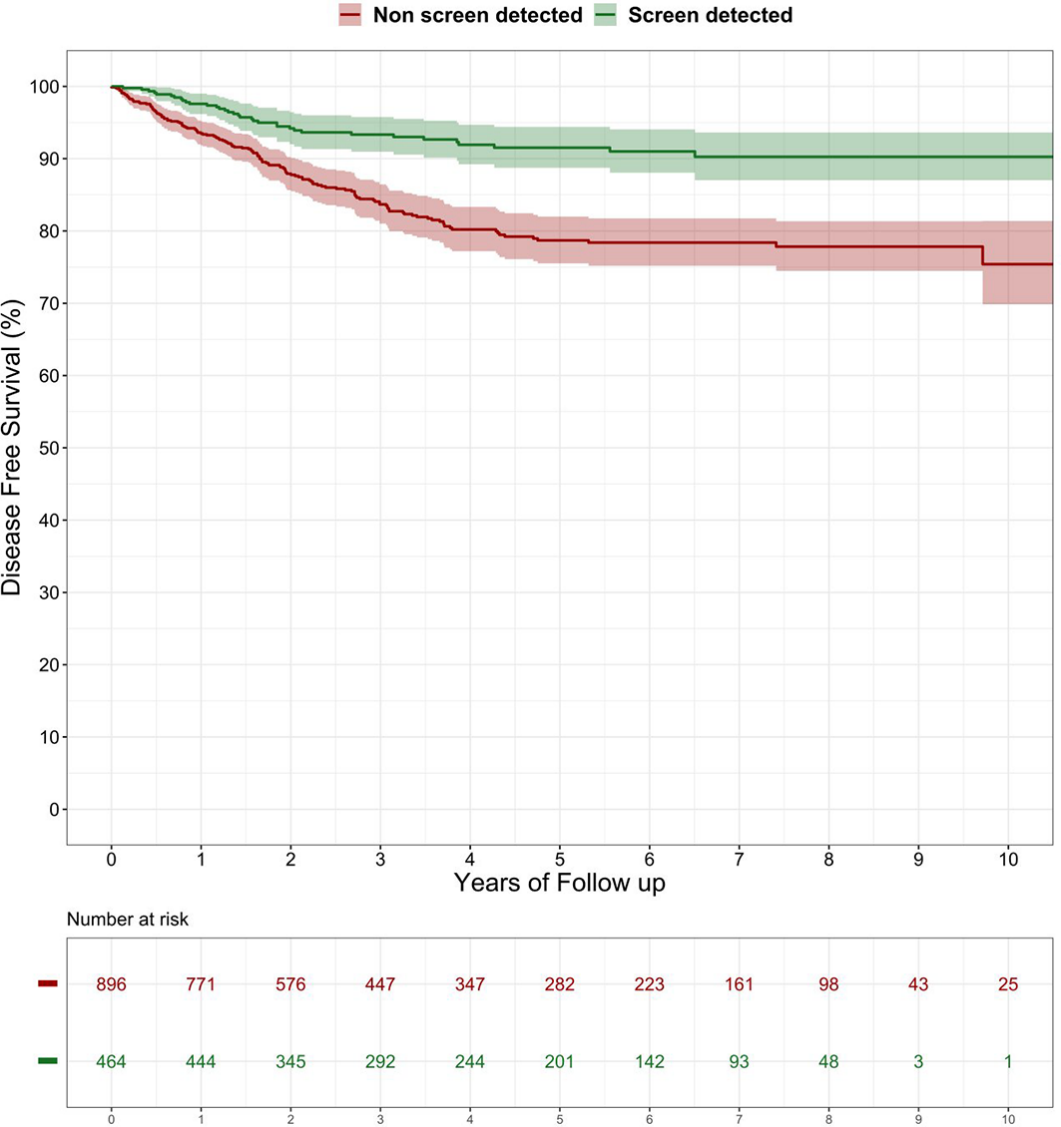
Overall survival after surgery among patients with screen detected versus non-screen detected colorectal cancer (%). Log-rank test: p-value < 0.0001.

Median follow up was 54.8 months. Overall, the 3- and 5-year DFS was 87.1% and 83.3%, respectively, and the 3- and 5-year OS was 86.2% and 79.5%, respectively. Compared with the SD, the NSD patients had a significantly lower DFS (log rank p < 0.0001) (**Figure 1**) and OS (log rank p < 0.0001) (**Figure 2**). The lower risk of death and recurrence among SD patients was confirmed after adjusting for demographic, histopathologic, and therapy-related variables [OS: Hazard Ratio (HR) 0.25, 95% CI 0.12–0.51; DFS: HR 0.40, 95% CI 0.22–0.73] (**Table 4**). This pattern was confirmed for Overall Survival of cases with TNM stage I-II (HR 0.23; 95% CI 0.05–0.99, p = 0.05) and stage III (HR 0.15; 95% CI 0.03–0.73, p = 0.02), while no significant differences were observed in terms of Disease Free Survival (stage I-II HR 0.44; 95% CI 0.16–1.20, p = 0.11; stage III HR 0.38; 95% CI 0.13–1.09, p = 0.07), possibly due to the low number of stage-specific events.

## Discussion

Colorectal Cancer Screening has been extensively implemented in Europe since 2003, when the European Council recognized the efficacy of FIT and recommended its employment as a method to detect CRC (10). In most Italian regions, FIT is proposed every two years to people aged 50–69 years old [apart from Piedmont, where Flexible Endoscopy (FS) represents the approach of choice (22)]. Several trials showed that SD cancers tend to have a better prognosis compared to NSD ones (14–16). This difference remains even when considering patients with advanced disease, and could be attributed to a number of factors, including a more favorable biology of SD tumors and a higher attention to health care of patients who decide to adhere to screening programs.

Our study aimed to evaluate the association between diagnostic modality (SD vs. NSD) and short- and long-term outcomes, as well as and on the quality of the therapeutic pathway of patients undergoing surgery for CRC.

The inclusion of short-term outcomes in our analysis was also due to the will to ascertain the impact of screening on appropriateness and quality of care, and consequently on the consumption of resources (for example, in case of readmission and major complications).

Our results concerning short-term outcomes are in line with those found in the literature, demonstrating an advantage for SD patients, even after adjusting the data for demographic, histopathologic, and therapy-related variables. As a possible explanation of this phenomenon, we considered the time from diagnosis to treatment, in the hypothesis that SD patients could have an easier access to surgical evaluation and treatment. Nonetheless, our hypothesis did not find confirmation in the statistical analysis: on the contrary, SD patients resulted to have a longer diagnosis to treatment time. These findings could be because, in the Veneto region, gastroenterologists almost exclusively conduct the diagnostic workup for FIT positives within screening programs; thus, SD patients have to wait for the result of histological examination to be referred to a colorectal surgeon, in order to continue the diagnostic and therapeutic process. Differently, a proportion of NSD patients receive their diagnosis directly by surgeons. Anyhow, this delay does not affect prognosis, since SD patients resulted to have all the same better short-term outcomes.

We also compared long-term DFS and OS of the two groups, thus showing that SD patients have a significantly better DFS and OS compared to NSD ones, as previously shown in other trials (3, 5). Again, this trend is confirmed after adjusting the data for clinical stage at diagnosis and, limited to OS, to stage specific analysis (TNM stage I-II, and III). The persistence of these results after adjustment by multiple histopathologic variables makes the association of the favorable prognosis of SD cases with less aggressive tumor biology unlikely. A residual intra-stage difference (which might not be entirely accounted for through adjustment by stage), or more organized clinical pathways for SD cases could explain the observed figures.

Another interesting finding, which could also be related to the better long-term outcomes observed in the SD group, concerns the higher percentage of screen-detected patients who received adjuvant chemotherapy within the “optimal” window. This could be because such patients are integrated in a pre-defined cure pathway, which ensures optimal collaboration among various specialists, including oncologists.

Recently, a trial by the Dutch ColoRectal Audit compared surgical outcomes of CRC patients diagnosed through the national screening program and patients with NSD CRC (17). The study included more than 53,658 patients, who underwent elective surgery for CRC between January 2011 and December 2016. Outcomes included postoperative complications, both surgical and non- surgical; complicated course (i.e., complications leading to a hospital stay of >14 days, a re-intervention and/or mortality); and 30 days mortality. The authors reported significantly better postoperative outcomes for SD patients, in line with previous results from literature; this difference, anyhow, did not subsist considering only rectal cancer patients.

Compared to the Dutch trial (17), our study also included patients treated in low-volume hospitals and is informative of the current clinical practice in Italy. We chose to include patients from peripheral centers in order to offer a more representative picture of the outcomes of CRC screening in the Veneto region. This is also due to the consideration that colorectal surgery is strongly decentralized, and many patients who are diagnosed with CRC are treated in second- or even first- level centers. Policies aimed at improving the quality of colorectal centers are ongoing in Italy (23), and recent studies showed favorable outcomes of colorectal surgery performed at peripheral hospitals, particularly for colon cancer (24).

This is multicentric study, which can be hold as representative of the results of CRC screening in a defined area (i.e., the Veneto region, Italy). Of course, several limitations need to be considered when interpreting our data. As stated before, the consistency of our data is limited by the inclusion of peripheral centers, also resulting in a high variability of cases provided by the single centers. Some centers provided data for a limited amount of time; however, no relevant changes were introduced during the study period in the management of CRC patients, so that the effect of this selection is likely marginal.

Besides, data concerning complications were not provided by surgical units, but derived from regional registries. Anyhow, this method has been validated in previous studies, and allows recognizing also complications not reported in the clinical datasets (25, 26). Furthermore, the retrospective nature of this study can be considered an important design limitation, even though it is counterbalanced by a prospective collection of data.

Our study considered patients aged 50–69 years. This could limit the generalizability of our results to countries where screening is proposed to different age groups. However, 50–69 years is by far the most commonly used target for FIT-based screening programs worldwide (11). Anyway, we are confident that our study allows to depict the Italian scenario and to answer our research question on the impact of screening among patients of a homogeneous age group.

One final limit of this study is that we could not account for the effect of overdiagnosis and length bias on the favorable outcomes observed among SD cases (27). However, the only study on overdiagnosis in CRC screening found a marginal risk of CRC overdiagnosis (below 0.4% for screening colonoscopies conducted on men younger than 75 years) (28). Since the sensitivity for CRC of FIT is lower than colonoscopy (29), the effect in our study is likely to be minimal. Differently, as far as we know, the size of length bias in CRC screening programs has never been estimated yet, and could actually represent a source of distortion of our results. Finally, our results in terms of long-term oncological outcomes may have been affected in favor of SD cases by lead-time bias. In fact, residual confounding could not be excluded even after adjusting the statistical models by stage at diagnosis.

In conclusion, screen detected CRC was associated to better short-term and long-term outcomes compared to non-screen detected CRC, and this difference remained after adjusting the results for patients’ clinic-pathological characteristics. Our results confirm the advantages for the target population to participate in the screening programs and comply with their therapeutic pathways.

## Data Availability Statement

The datasets presented in this article are not readily available because datasets will be made available upon reasonable request to the corresponding author. Requests to access the datasets should be directed to gaya.spolverato@gmail.com.

## Ethics Statement

Ethical review and approval was not required for the study on human participants in accordance with the local legislation and institutional requirements. Written informed consent for participation was not required for this study in accordance with the national legislation and the institutional requirements.

## Author Contributions

GS, SP, MZ, and MR: conception and design. All authors recruited patients, performed procedures, and gathered data. GS, MZ, JB, GC, AB, and SP: data analysis and interpretation. GS, MZ, JB, GC, AB, and SP: drafting of the article. All authors gave critical revision of the article for important intellectual content. All authors contributed to the article and approved the submitted version.

## Conflict of Interest

The authors declare that the research was conducted in the absence of any commercial or financial relationships that could be construed as a potential conflict of interest.
