# Supplementary material for: More Favorable Short and Long-Term Outcomes for Screen-Detected Colorectal Cancer Patients
Source: Front Oncol. 2021 Mar 15;11:620644. doi: 10.3389/fonc.2021.620644 (PMC8005726; doi:10.3389/fonc.2021.620644)
Supplement: Supplementary file 2 [file DataSheet_2.pdf]

SUPPLEMENTARY MATERIAL

Supplementary Table 1. Adjusted Odds Ratios of the quality of the therapeutic pathway and of short-term outcomes, and adjusted Hazard Ratios of Disease Free- and Overall Survival by diagnostic modality, with 95% Confidence Intervals (CI) for colonic cancer

Category	Outcome	Adjusted[1] Odds Ratio (SD vs NSD) (95% CI)	p-value
1. Quality of the therapeutic pathway	1.1: Time between diagnosis and treatment ≤ 30 days	0.46 (0.30-0.70) [1]	0.0003
	1.2: Time between surgery and chemotherapy < 8 weeks	2.17 (1.05-4.48) [2]	0.03
	1.3: Length of postoperative hospital stay ≤ 12 days	1.30 (0.64-2.61) [2]	0.47
2. Short-term outcomes	2.1: 30 days readmission	0.33 (0.12-0.93) [3]	0.03
	2.2: 30 days surgery- related readmission	0.32 (0.09-1.06) [3]	0.06
	2.3: 30 days reintervention	0.56 (0.26-1.21) [3]	0.14
	2.4: 30 days surgery- related reintervention	1.12 (0.33-3.80) [3]	0.86
	2.5: EBL >500mL	0.26 (0.08-0.79) [3]	0.02
	2.6: Readmission due to surgical complications	0.36 (0.06-2.21) [3]	0.27
	2.7: Readmission due to medical complications	0.60 (0.10-3.66) [3]	0.58
	2.8: Reinterventions due to surgical complications	0.35 (0.08-1.56) [3]	0.17
	2.9: Reinterventions due to medical complications	0.55 (1.11-2.83) [3]	0.48
	2.10: Surgical complications	0.49 (0.23-1.00) [3]	0.05
	2.11: Medical complications	0.68 (0.28-1.63) [3]	0.38
	2.12: Complications (surgical or medical)	0.54 (0.29-0.99) [3]	0.04
3. Survival	3.1: Disease Free Survival	0.37 (0.17-0.81) [4]	0.01
	3.2: Overall Survival	0.32 (0.14-0.72) [4]	0.0059

[1] according to logistic regression adjusted by gender, age, CRC stage at diagnosis, surgery unit, comorbidity

[2] according to logistic regression adjusted by gender, age, CRC stage at diagnosis, surgery unit, comorbidity, surgical approach, stoma creation and neoadjuvant treatment

[3] according to logistic regression adjusted by gender, age, CRC stage at diagnosis, surgery unit, comorbidity, surgical approach, stoma creation, neoadjuvant treatment, resection margin, lymphatic invasion, vascular invasion, perineurial invasion, grading

[4] according to Cox regression model adjusted by gender, age, CRC stage at diagnosis, surgery unit, comorbidity, surgical approach, stoma creation, neoadjuvant treatment, resection margin, lymphatic invasion, vascular invasion, perineurial invasion, grading, adjuvant treatment

SD= screen detected; NSD=non screen detected; EBL= Estimated Blood Loss

Supplementary Table 2. Adjusted Odds Ratios of the quality of the therapeutic pathway and of short-term outcomes, and adjusted Hazard Ratios of Disease Free- and Overall Survival by diagnostic modality, with 95% Confidence Intervals (CI) for rectal cancer.

Category	Outcome	Adjusted[1] Odds Ratio (SD vs NSD) (95% CI)	p-value
1. Quality of the therapeutic pathway	1.1: Time between diagnosis and treatment ≤ 30 days	0.71 (0.41-1.24) [1]	0.23
	1.2: Time between surgery and chemotherapy < 8 weeks	3.02 (0.83-11.03) [2]	0.42
	1.3: Length of postoperative hospital stay ≤ 12 days	1.08 (0.49-2.40) [2]	0.84
2. Short-term outcomes	2.1: 30 days readmission	2.19 (0.57-8.40) [3]	0.25
	2.2: 30 days surgery- related readmission	3.67 (0.81-16.71) [3]	0.09
	2.3: 30 days reintervention	0.79 (0.25-2.50) [3]	0.69
	2.4: 30 days surgery- related reintervention	0.51 (0.11-2.33) [3]	0.38
	2.5: EBL >500mL	0.03 (<0.001-3.23) [3]	0.15
	2.6: Readmission due to surgical complications	3.50 (0.48-25.70) [3]	0.22
	2.7: Readmission due to medical complications	1.02 (0.12-8.65) [3]	0.98
	2.8: Reinterventions due to surgical complications	0.81 (0.15-4.35) [3]	0.81
	2.9: Reinterventions due to medical complications	0.27 (0.01-7.05) [3]	0.43
	2.10: Surgical complications	1.65 (0.53-5.19) [3]	0.39
	2.11: Medical complications	2.37 (0.36-15.74) [3]	0.37
	2.12: Complications (surgical or medical)	2.35 (0.81-6.81) [3]	0.11
3. Survival	3.1: Disease Free Survival	0.63 (0.19-2.17) [4]	0.47
	3.2: Overall Survival	0.01 (0.00-0.41) [4]	0.02

[1] according to logistic regression adjusted by gender, age, CRC stage at diagnosis, surgery unit, comorbidity

[2] according to logistic regression adjusted by gender, age, CRC stage at diagnosis, surgery unit, comorbidity, surgical approach, stoma creation and neoadjuvant treatment

[3] according to logistic regression adjusted by gender, age, CRC stage at diagnosis, surgery unit, comorbidity, surgical approach, stoma creation, neoadjuvant treatment, resection margin, lymphatic invasion, vascular invasion, perineurial invasion, grading

[4] according to Cox regression model adjusted by gender, age, CRC stage at diagnosis, surgery unit, comorbidity, surgical approach, stoma creation, neoadjuvant treatment, resection margin, lymphatic invasion, vascular invasion, perineurial invasion, grading, adjuvant treatment

SD= screen detected; NSD=non screen detected; EBL= Estimated Blood Loss
